# Polylactide-Based Stent Coatings: Biodegradable Polymeric Coatings Capable of Maintaining Sustained Release of the Thrombolytic Enzyme Prourokinase

**DOI:** 10.3390/ma12244107

**Published:** 2019-12-09

**Authors:** Alexander S. Baikin, Alexey G. Kolmakov, Lyudmila A. Shatova, Elena O. Nasakina, Mars G. Sharapov, Ilya V. Baymler, Sergey V. Gudkov, Mikhail A. Sevostyanov

**Affiliations:** 1 Baikov Institute of Metallurgy and Materials Science of the Russian Academy of Sciences, Leninsky Prospekt 49, Moscow 119334, Russia; imetranlab10@mail.ru (A.G.K.); nacakina@mail.ru (E.O.N.); cmakp@mail.ru (M.A.S.); 2 Institute of Strength Physics and Materials Science of the Siberian Branch of Russian Academy of Sciences, Tomsk 634055, Russia; 3 Department of Physics, Voronezh State Technical University, st. 20-letiya Oktyabrya, 84/4, Voronezh 394006, Russia; shatovala@mail.ru; 4 Institute of Cell Biophysics of the Russian Academy of Sciences, 3 Institutskaya St., Pushchino, Moscow Region 119991, Russia; 5 Moscow Institute of Physics and Technology (National Research University), Institutsky Lane 9, Dolgoprudny, Moscow Region 141700, Russias_makariy@rambler.ru (S.V.G.); 6 Prokhorov General Physics Institute of the Russian Academy of Sciences, 38 Vavilova St., Moscow 119991, Russia; 7 Institute of Biology and Biomedicine, Lobachevsky State University of Nizhny Novgorod, 23 Gagarin Ave., Nizhny Novgorod 603950, Russia

**Keywords:** coatings, stent, biodegradable, polymeric, polylactide, maintaining sustained release

## Abstract

The novelty of the study is the development, creation, and investigation of biodegradable polymeric membranes based on polylactide, that are capable of directed release of large molecular weight biomolecules, particularly, prourokinase protein (MW = 46 kDa). Prourokinase is a medication with significant thrombolytic activity. The created membranes possess the required mechanical properties (relative extension value from 2% to 10%, tensile strength from 40 to 85 MPa). The membranes are biodegradable, but in the absence of living cells in a water solution they decompose by less than 10% in half a year. The created membranes are capable of controlled prourokinase release into intercellular space, and the total enzymatic activity of prourokinase does not decrease by more than 12%. The daily release of prourokinase from one square centimeter of the membrane ranges from 1 to 40 μg per day depending on the technique of membrane preparation. The membranes have no acute toxic effect on cells accreting these surfaces de novo. The number of viable cells is at least 96%−97% of the overall cell count. The mitotic index of the cells growing on the surface of the polymeric films comprised around 1.5%. Histological examination did not reveal any disorders in tissues of the animals after the implantation of polymer membranes based on polylactide, both alone and as components of stent cover. Implantation of stents covered with prourokinase-containing polymers led to the formation of a mature connective tissue capsule that is thicker than in the case of uncovered stents. Thus, various polylactide-based biodegradable polymeric membranes possessing the required mechanical properties and capable of prolonged and directed release of prourokinase macromolecules are developed and investigated in the study.

## 1. Introduction

The creation of the controlled release and targeting of pharmaceutical agents based on biodegradable polymers is one of the most promising and rapidly growing fields in chemical technology [1]. One of the possible areas of application for systems of controlled release and targeting of pharmaceutical agents is their use in coatings of medical devices, especially implants [2]. The local release of medications from biodegradable coverings of implants can solve a number of post-operative complications in the site of implantation. In the case of stents, these are the prevention of restenosis, thrombosis, and so forth [3]. For more massive implants it is, first of all, the prevention of the inflammatory reactions [4]. One of the major tasks in the creation of biodegradable polymers for covering the implants is the elevation of the biocompatibility degree of the medical device as a whole [5]. The current study focuses right on this actual issue. One of the promising materials for the development of controlled pharmaceutical agent targeting systems is polylactide [6]. Polylactide is an aliphatic polyester, which is produced by the ring-opening polymerization of lactide. Technological approaches used in the production allow to change the physic-chemical properties of the polymer (chain length, degree of branching) [7]. It is known that a lactic acid polycondensation reaction can yield only a low molar mass polylactide. The product of polycondensation is subjected to chemical treatment and then polymerized, yielding a high molar mass polylactide [8]. A high molar mass polylactide is biodegradable and thermoplastic. An obvious advantage of polylactide is the use of renewable resources for its production, such as silage crops. Polylactide-based polymer films can be used in the creation of dosage formulations [9], in encapsulation technologies [10], sorption [11], target therapy [12], and so forth. Currently, polylactide is already used in healthcare in the production of surgical sutures [13], implants [14], and in regenerative therapy to repair the defects of bone and cartilage tissues [15]. Searching and testing new polylactide-based polymeric products is still in progress.

The principal possibility of the creation of a system of controlled targeting of low molecular weight compounds on base of polylactide is well-known [16]. However, for high molecular weight compounds, such a polylactide-based system that possesses all the required physicochemical properties has not been shown to exist. The current work shows technological solutions required to obtain biodegradable polymer membranes and polylactide-based coatings with needed mechanical properties. The goal of the current work was to create polymeric biodegradable coverings for stents capable of the prolonged release of a high-molecular weight anti-thrombosis agent, prourokinase. Prourokinase is a single-chain protein molecule with a molecular weight of 46 kDa composed of two domains, 17 kDa and 29 kDa, which contain regulatory and catalytic parts of the enzyme, respectively, and are linked by a disulfide bridge [17]. Due to its regulatory part, prourokinase specifically interacts with fibrin-bound plasminogen and catalyzes plasminogen conversion to plasmin, a protease capable of lysing fibrin clots (thrombi). Today, prourokinase is an effective, cheap, and available compound for thrombolytic therapy [18]. The principal novelty of the presented study is the creation and investigation of various biodegradable polylactide-based polymeric membranes possessing the required mechanical properties and capable of a prolonged and targeted release of biomacromolecules of high molecular weight.

## 2. Materials and Methods

### 2.1. Polylactide Membrane Preparation

**Figure 1:**
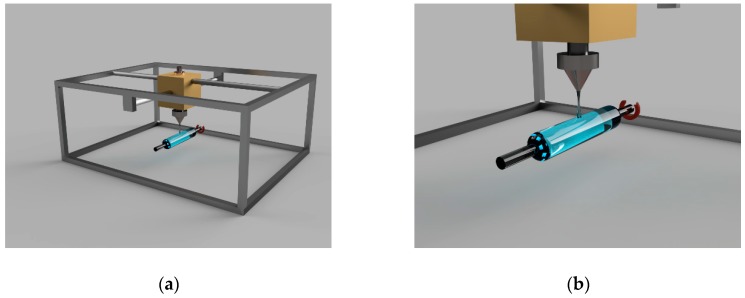
A device for applying polymer to the surface of stents: (**a**) General view of the device; (**b**) general view of the polymer coating head and wire with polymer. The arrow indicates the direction of movement of the wire.

An amorphous polylactide (D, L-LA,) with a molar mass of 45, 90, and 180 kg/mol was used in the experiments. Molar masses are determined by the manufacturer of polylactide (Nature Work, Minnetonka, MN, USA). The initial experiments demonstrated the most promising range of polylactide solutions concentrations to be from 2.8% to 3.2%. Three percent polylactide solution in chloroform (Irea2000, Moscow, Russia) was prepared by mixing to homogeneity during 1 h at 57 °C. Colloidal solution of prourokinase in PBS (15 mM, pH = 7.4) was added to the homogenous solution for membrane deposition. The resulting solution in 12 mL portions was poured into plastic forms (85 mm in diameter). The polylactide membranes were obtained by the casting of polymer solution with a subsequent two-step evaporation of the solvent. The first step was carried out during 3 h at 50 Torr, the second drying step took 5 h at 3 Torr. The membranes were deposited after congelation for 24 h at 40 °C. To apply polymers onto a wire for stents, a three-component appliance was constructed. The first part was a multi-axial manipulator for motion of the actuator; the second part was an actuator for applying polymer onto wire (extruder with an ultrasonicator); the third part was a system for drawing and rotating the wire (Figure 1).

### 2.2. Examination of Mechanical Properties of Polylactide Films

The tensile strength of the polylactide films (pure and drug-infused) was assessed on a universal testing machine INSTRON 3382 (Instron Corp., Norwood, MA, USA) at the loading rate of 10 mm/min. Film samples (100 µm thick; in the shape of a double spade) were fixed in the grips of the testing machine, which were tightened evenly to ensure that samples did not slip during tests. The morphology of the sample surface was examined using a scanning electron microscope (SEM) TESCAN VEGA II SBU (TESCAN, Brno-Kohoutovice, Czech Republic).

### 2.3. Release of Prourokinase from Polylactide Films and Dissolution of the Polymer

The optical spectra of polylactide and prourokinase aqueous solutions differ substantially. This fact was used as a basis for the spectroscopic monitoring of changes in the prourokinase and polylactide concentrations in aqueous systems. The changes were comparatively monitored in the systems of “prourokinase + polymer + water” versus “polymer + water” and “polymer + water” versus “water” [19]. Absorbance of the solutions was measured using a spectroscopic system USB 2000 (Ocean Optics, Largo, FL, USA). Prourokinase absorbance was measured at 280 nm; it varied in the range of 0.1−2.5, with the mass absorption coefficient of the enzyme being equal to 0.133 L/g. In addition to being measured spectroscopically, the dissolution of polylactide was also assessed by weighing the dry residue after evaporation of samples. The measurements of prourokinase release were accompanied by the measurements of the enzyme activity.

### 2.4. Determination of the Proteolytic Activity of Prourokinase

Aliquots of samples were normalized with respect to protein concentration (A280 nm = 0.1) and analyzed for amidolytic activity on Glu-Gly-Arg-pNA (S-2444; Chromogenix, Mölndal, Sweden) as described previously [20]. Activity of uPA (10 ng/mL) in solution in 50 mM Tris (tris(hydroxymethyl)aminomethane)-HCl, 100 mM NaCl, pH 7.4, at 37 °C was studied by continuous assay using S-2444. uPA and S-2444 were equilibrated at 37 °C in the spectrophotometer sample cuvette. The absorbance at 406 nm was monitored over time. Absorbance measurements were transformed using the first derivative function to yield S-2444 (0.3 mM) hydrolysis velocities (ΔA406/Δt).

### 2.5. Cell Culture Studies

The studies of the polymer biocompatibility were performed using standard in vitro test systems. A culture of human neuroblastoma cells (SH-SY5Y) was used as a standard experimental model. The SH-SY5Y cell line was subcloned from the line SK-N-SH, which was isolated from the bone marrow material of a four-year-old neuroblastoma patient [17]. The SH-SY5Y cell line is a classical model for neuronal functions and differentiation in vitro. In tissue cultures, SH-SY5Y cells usually develop in two different ways, which makes them useful in the study of cell differentiation. Additionally, cells of the SH-SY5Y line are interesting because of their ability to form, not only monolayers, but also aggregates, which are able to adhere to the substrate. Moreover, when growing in vitro, SH-SY5Y cells can spontaneously switch between two phenotypes: neuroblastomic and epithelial [21]. SH-SY5Y cells were cultivated in a DMEM medium (Biolot, St. Petersburg, Russia) supplemented with 10% fetal calf serum (Gibco, Gaithersburg, MD, USA) and 30 µg/mL gentamicin in a CO_2_ incubator (Binder, Tuttlingen, Germany) at 37 °C and 5% CO_2_. Samples of polylactide films (20 × 20 mm) were placed in 35 mm Petri dishes (1 sample per dish), followed by inoculation with SH-SY5Y cells (10^4^ cells/cm^2^; 3 mL per dish). After cultivation for 3 days, the cells growing on the surface of the polymer samples were stained with the fluorescent dyes Hoechst 33342 (Sigma, St. Louis, MO, USA; 2 µg/mL) and propidium iodide (Sigma, St. Louis, MO, USA; 2 µg/mL). Hoechst 33342 stains both alive and dead cells. Propidium iodide practically does not stain alive cells within the time frame of the staining procedure (about 10 min); it only penetrates into the cells whose plasma membrane is damaged, which is a characteristic feature of dead cells. After staining the polymer samples, the number of alive and dead cells on their surface was counted using an imaging system Leica DMI6000 (Leica, Munich, Germany). At least 500 cells in total were counted per each sample [22]. The number of cells undergoing mitosis was counted under a fluorescence microscope using the technique of vital staining with Hoechst 33342 (Sigma, St. Louis, MO, USA). Mitotic cells were identified by the distribution of chromatin characteristic of prophase (P), metaphase (M), anaphase (A), and telophase (T). At least 500 cells in total were counted per each sample. The mitotic index (MI) was calculated using the formula MI = (P + M + A + T)/N · 100%, where (P + M + A + T) is the number of cells at different mitotic phases and N is the total number of analyzed cells [23].

### 2.6. Experimental Animals

In the experiments, male Wistar rats (180−200 g) were used. The animals were kept under standard conditions (temperature, 22 ± 2 °C; humidity, 30%−70%; 12 h light period; feed and water ad libitum). All animal manipulations were approved by the Bioethics Committee of the Institute of Theoretical and Experimental Biophysics of the Russian Academy of Sciences. At least 5 animals were used to study each material sample.

### 2.7. Implantation Tests

For the assessment of biocompatibility of polylactide films, a rat model of subcutaneous biomaterial implantation was used [24]. To sterilize the film surface and prevent the primary acute reaction of the recipient organism, the polymer samples were conditioned for 4 h at 37 °C in a nutrient medium containing 10% cattle serum, a mixture of antibiotics, and an antimycotic. Samples were labeled with colored silk threads and implanted subcutaneously by blunt dissection through a cut in the lower part of the back using a trocar. The samples were implanted along the back line of the animal (3 samples per rat; spaced 3 cm from each other). The surgery was carried out under Zoletil/xylazine anesthesia (6/12 mg per 1 kg of body weight). The operated animals were sacrificed 60 days after the implantation [25].

## 3. Results

**Figure 2:**
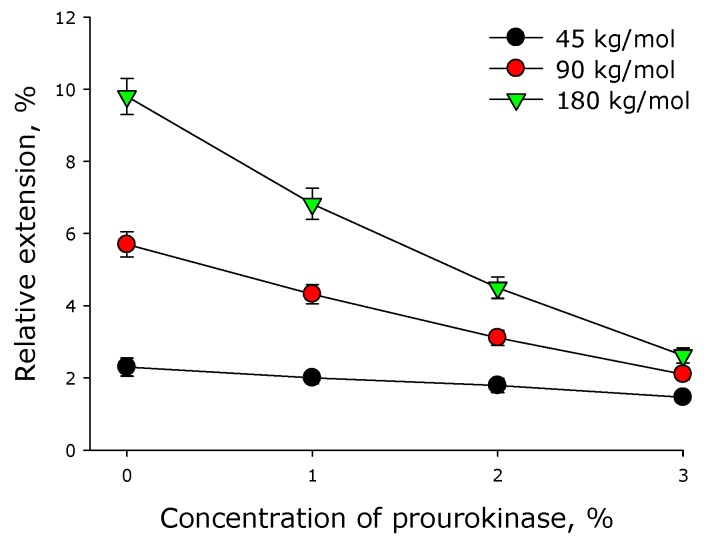
Effect of prourokinase on the elongation of polymer films made of polylactide of different molar mass. Mean values and their standard errors calculated from the data of three independent experiments are given.

As shown in Figure 2, the molar mass of polylactide, as well as the content of prourokinase in the polymer film, substantially affected the value of percentage elongation of the film. The incorporation of prourokinase into a polylactide film reduced its ability to elongate. In general, the elongation of the polylactide films examined linearly depended on the content of prourokinase. The elevation of prourokinase concentration by 1% caused the decrease of a specific extension by 10% in 45 kg/mol polylactide polymer, by 20% in 90 kg/mol polylactide polymer, and by 25% in 180 kg/mol polymer.

**Figure 3:**
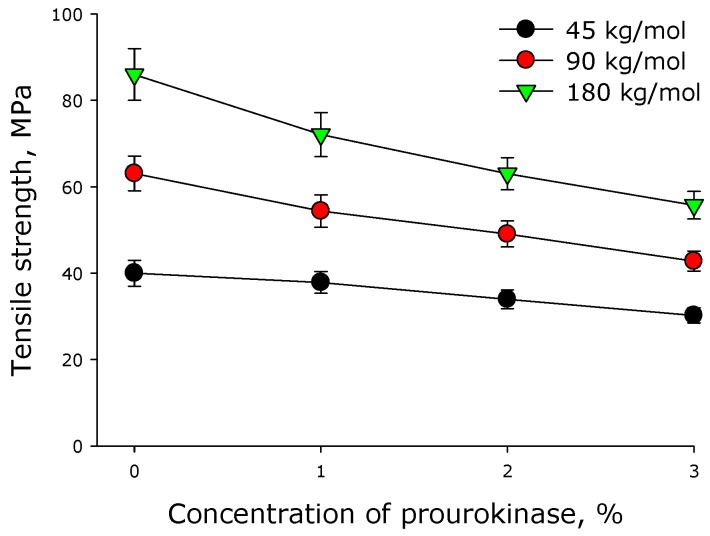
Effect of prourokinase on the tensile strength of polymer films made of polylactide of different molar mass. Mean values and their standard errors calculated from the data of three independent experiments are given.

Figure 3 shows the effect of prourokinase on the tensile strength of polymer films made of polylactide of different molar mass. As it turned out, the introduction of prourokinase in the polymer films decreased their tensile strength. Generally, tensile strength of the films depends linearly on the concentration of prourokinase. A 1% increase in the concentration of prourokinase diminished the tensile strength of a polylactide film by 15%−20%. The use of a high-molecular polylactide increased the ultimate strength of the polymer films. For example, the ultimate strength of the films made of polylactide with the molar mass 45 kg/mol was more than twice as low as that of the films made of 90 kg/mol polylactide. The latter films were, in their turn, 30% less strong than the films made of 180 kg/mol polylactide.

**Figure 4:**
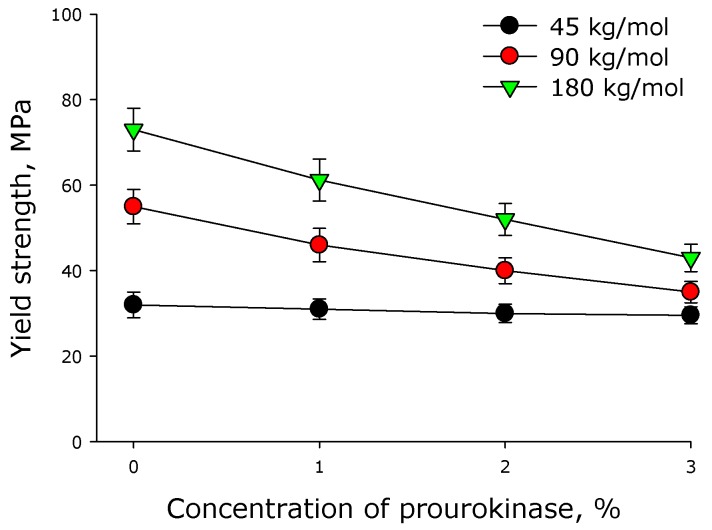
Effect of prourokinase on the yield strength of polymer films made of polylactide of different molar mass. Mean values and their standard errors calculated from the data of three independent experiments are given.

Figure 4 shows the yield strength of polylactide films versus the concentration of prourokinase. One can see that the higher the molar mass of polylactide is, the higher the yield strength of the polymer film. Incorporation of prourokinase into a film results in the reduction of its yield strength.

**Figure 5:**
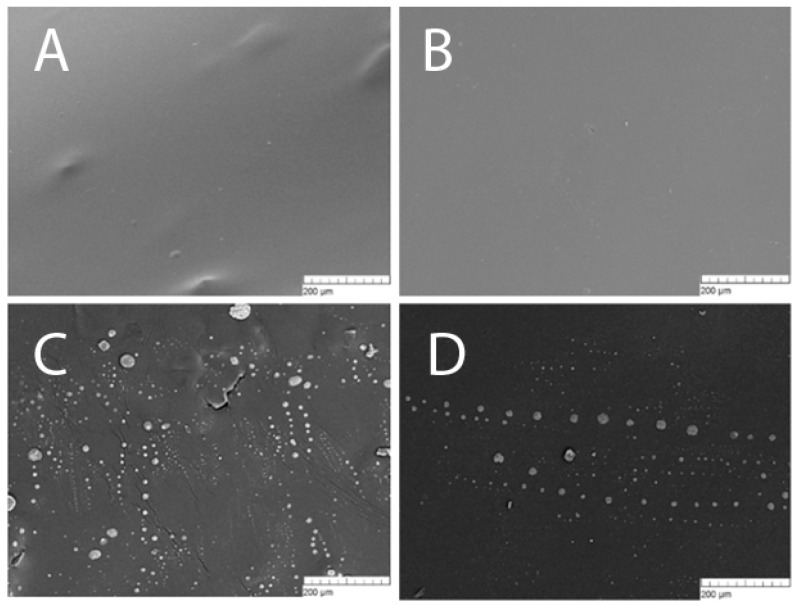
Micrographs of the surface of 45 kg/mol polylactide films without prourokinase (**A**) and with 1% (**B**), 2% (**C**), and 3% (**D**) prourokinase. The micrographs were obtained using a scanning electron microscope, at a magnification of ×300.

Represented in Figure 5 are micrographs of the surface of polylactide films, either with or without prourokinase at different concentrations. The images demonstrate that the technology we used yields films with smooth surfaces, which are free of any defects. At higher concentrations of prourokinase (2%−3%), there might be protein aggregates and dried droplets of saline on the surface of films, which was quite a rare occurrence observed in some film batches. Such protein aggregates mostly appeared on the surface of films made of polylactide with the molar mass of 45 kg/mol. For the films made of high-molecular polylactides, that was not a problem.

**Figure 6:**
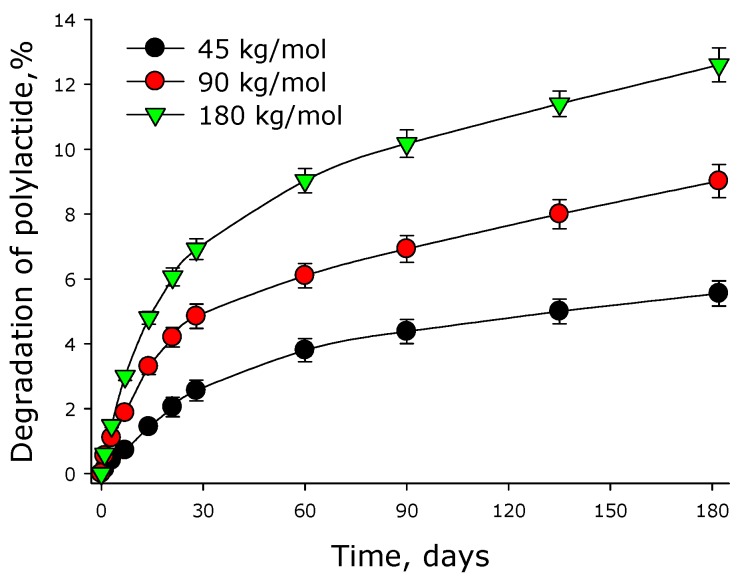
The rate of decomposition of polylactide films depending on the molar mass of the polymer. Mean values and their standard errors calculated from the data of three independent experiments are given.

Figure 6 shows the dependence of the rate of decomposition of polylactide films on the molecular mass of the polymer. One can see that the lower the molecular mass of polylactide is, the slower the degradation of films made of this polymer. The degradation of polylactide was found to occur in two phases. The first phase took about one month from the moment a film was put into a saline solution for storage and was characterized by a relatively high rate of degradation. During the second phase, which began after storing a film for about a month, the rate of film degradation dropped substantially. For the films made of 45 kg/mol polylactide, the degradation rate at the first phase was about 0.09% of polymer weight per day—and it became three times as low during the second phase (0.03% of polymer weight per day). For 90 kg/mol polylactide films, the values were 0.18% and 0.04% of polymer weight per day, respectively; for 180 kg/mol films, they were 0.25% and 0.05%—five times lower at the second phase in both cases. In a number of experiments, the degradation of polylactide films was monitored in phosphate buffers of different capacities and pH. As it turned out, both these parameters—buffer capacity and pH (7.0−8.0)—did not have a particular effect on the rate of film degradation, which was about the same as in saline. The presence of prourokinase in the polymer films increased the rate of their degradation by 15%−25%, yet the dynamics of degradation was very close to that of films without prourokinase.

**Figure 7:**
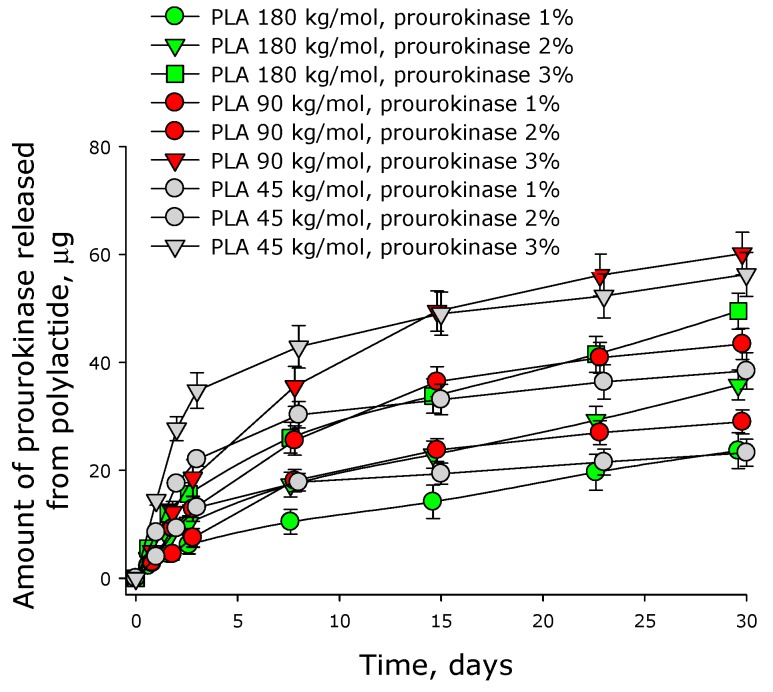
Dynamics of prourokinase release from polylactide films. Mean values and their standard errors calculated from the data of three independent experiments are given.

Our experiments showed that prourokinase was slowly released from 45 kg/mol polylactide films (Figure 7). The rate of the release depended on the initial concentration of the enzyme in the film. The higher the concentration was, the faster the enzyme diffused in the medium. For the films containing 1%, 2%, and 3% prourokinase, the initial rates of the enzyme release were about 12, 22, and 34 µg/day respectively. In other words, the initial rate of the prourokinase release was proportional to its concentration in the film. After 3 days, the rate of prourokinase release from polylactide films dropped substantially. When the initial concentration of the enzyme was 3%, the release of the enzyme practically stopped by the 4th day. At that point, the enzyme molecules remaining in the film (approximately 50% of the initial amount) were released in parallel with the dissolution of the film. For the films with the initial concentration of prourokinase 1% and 2%, the rates of the enzyme release also dropped—to the levels of 0.5 µg/day. By the 30th day, films with the initial content of prourokinase 1% only 10% of the protein remained (i.e., 90% of it had already been released). The molecules of prourokinase released from polylactide films retained over 90% of their enzymatic activity, indicating the preservation of the native protein packing in the films.

In the experiments with 90 kg/mol films, we also observed a slow release of prourokinase from the membrane (Figure 7). The rate of the release depended on the initial concentration of the enzyme in the film. The higher the concentration was, the faster the enzyme diffused in the medium. For the films containing 1%, 2%, and 3% prourokinase, the initial rates of the enzyme release were about 2, 4 and 6 µg/day respectively. In other words, the initial rate of prourokinase release was proportional to its concentration in the film. After 3 days, the rate of prourokinase release from polylactide films dropped substantially. When the initial concentration of the enzyme was 3%, the release of the enzyme practically stopped by the 8th day. At that point, the enzyme molecules remaining in the film (approximately 50% of the initial amount) would be released in parallel with the dissolution of the film.

Prourokinase was also found to diffuse from 180 kg/mol polylactide films (Figure 7). The dynamics of the enzyme release was similar to that observed with the films made of 45 and 90 g/mol polylactide. The initial rates of prourokinase release were about 1.5, 3, and 5 µg/day in case of films containing 1, 2, and 3% prourokinase, respectively. After 3 days, the rate of prourokinase release did not change substantially. Around 70% of prourokinase molecules resided in the membrane would probably be released from the membrane further with the set rate, another part would apparently be effluxed into the solution with the rate equal to polymeric membrane dissolution rate.

**Figure 8:**
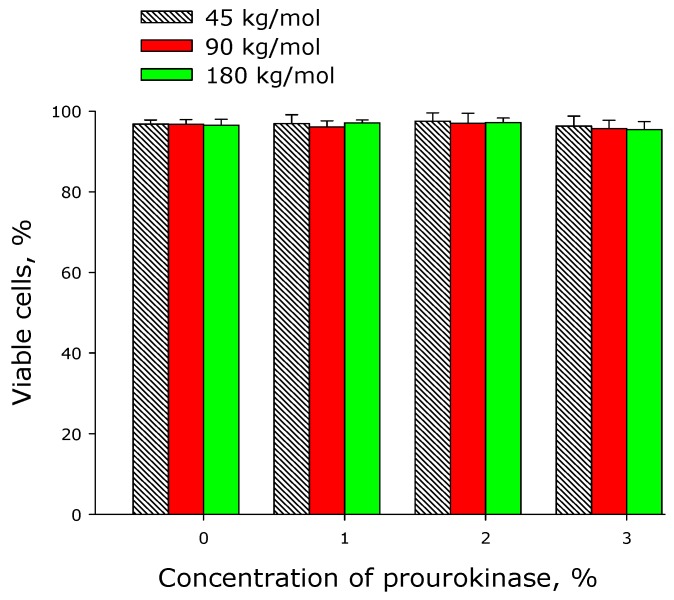
Effect of prourokinase-containing polylactide films on the viability of cell cultures. Mean values calculated from the data of three independent experiments are given.

Figure 8 shows the effect of polylactide films on the viability of cell cultures. The number of nonviable cells on the surface of prourokinase-free polylactide films did not exceed 3%−4%, which was even lower than the number of nonviable cells on the dish surface (5.5% ± 1.2%). When prourokinase is added to the polymer, there is no tendency for more non-viable cells to appear. In all cases, the films did not have any short-term toxic effects on the cells growing de novo on the sample surfaces.

**Figure 9:**
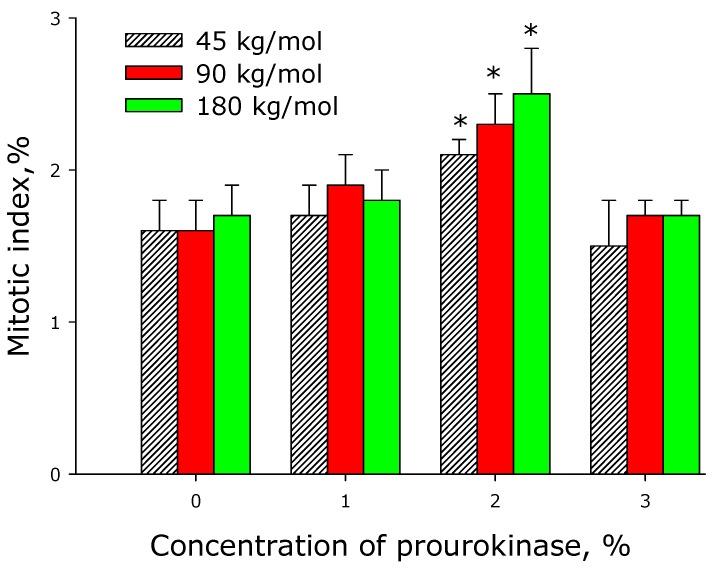
Effect of prourokinase-containing polylactide films on the mitotic index of cell cultures. Mean values calculated from the data of three independent experiments are given.

To analyze the mitotic activity of cells, we determined their mitotic index (MI) when the cells were in the phase of logarithmic growth (3rd day since the start of cultivation; Figure 9). The MI of cells growing on the surface of prourokinase-free films was approximately 1.6%. The incorporation of prourokinase (1 or 3%) into the films did not change the MI much. The concentration of prourokinase up to 2% led to the increase of the mitotic index of 2.0%−2.5%.

**Figure 10:**
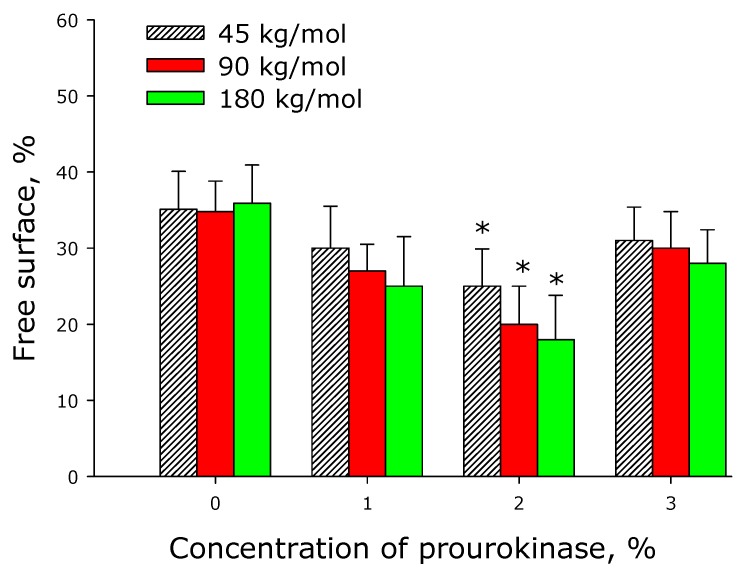
Populating surfaces of polylactide films with cells depending on the molar mass of the polymer and concentration of prourokinase. The averaged data of three independent experiments are given.

Figure 10 shows data on the spread of cells over available polymer surfaces by the 3rd day of cultivation. On prourokinase-free films, approximately 35% of their area remained unpopulated—just the same as on glass surfaces in control experiments. The same picture was observed when the films were infused with 1% or 3% prourokinase, 2% concentration of prourokinase in the polymer led to apparent free area around 20−25%. Analogous results were obtained in the study of polymer coatings with an antithrombotic agent applied to stents.

**Figure 11:**
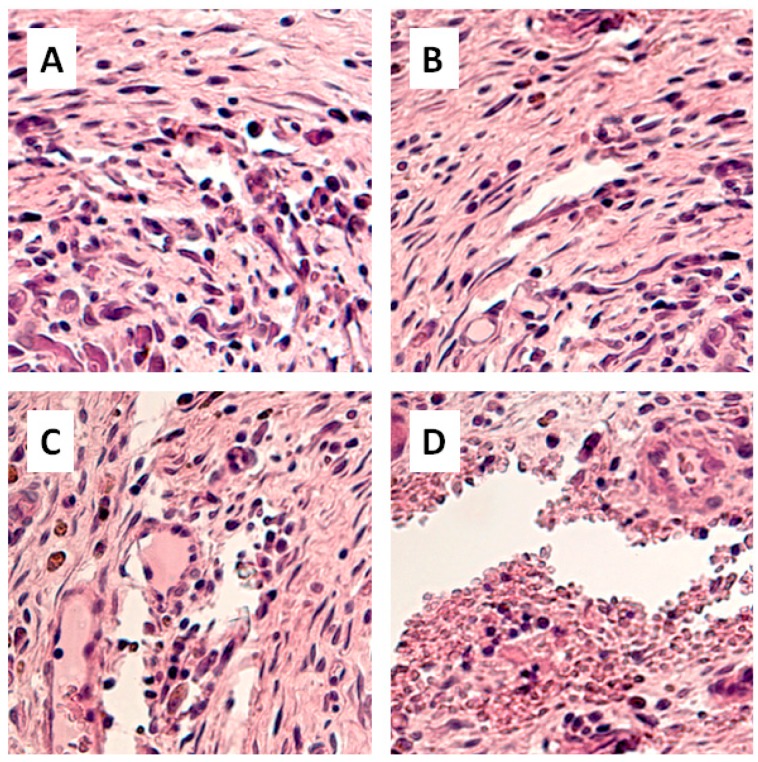
Sections of the tissues surrounding the prourokinase-containing films made from polylactide. The concentration of prourokinase in the polymer was 2%. The sections were obtained 8 weeks after the subcutaneous implantation of films in rats. Representative photographs of histological preparations are shown in sub-caption (**A**–**D**).

The biocompatibility of prourokinase-containing polylactide films was assessed in the experiments in vivo, in which films were implanted subcutaneously into the spinal muscle tissues of rats. In the postoperative period, the condition of all the animals was satisfactory, and they were removed from the experiment 60 days after surgery. The tissues surrounding implants were examined microscopically. The examination showed that tissue samples had no traces of prourokinase-containing polylactide (Figure 11A,B). In some cases, there were channels, which were left by the threads used to label the implants and flag the postoperative topology (Figure 11C,D). When the polymer films were used as coatings for Nitinol stents, the material was explanted and studied macroscopically. Only traces of polymers were found on the stent surface, and mature connective-tissue capsules were formed on the periphery of the samples. The capsules were characterized by a predominance of extracellular over cellular components, a regular arrangement of fibers, and the absence of vessels. These signs indicated a partial involution of the capsules around the implanted fragments. The structure and thickness of the capsules were standard for non-resorptive samples of this type, indicating bioinertness and biocompatibility of the fragments in general

## 4. Discussion

Mechanical properties were determined for all the created polymeric coverings, including the assessment of effect of prourokinase concentration onto specific extensions of polymer films of various molar mass polymers (Figure 2). The change of a specific extension for polylactide polymers of different molar masses has a linear character, all the straight lines intersecting in the neighborhood of a point {6% prourokinase concentration; 2% specific extension}, so, the lower concentrations of the enzymes make different specific extension values possible. Using the theory of elasticity of rubber, one considers a polymer chain in a cross-linked network as an entropy spring. When the chain stretches, entropy decreases with a large margin because fewer conformations are available [26]. It can be assumed that the presence of protein can affect the conformations of chains, because if they are more stretched in undeformed material, their ability to stretch is lower. Other mechanical properties of polylactide polymers were also established (Figure 3 and Figure 4). It shown that with the increasing protein concentration in the polymer, the ultimate tensile strength decreases. This may be due to mechanical weakness forming on the protein-PLA interface. With an increase in the molar mass of the polymer, the ultimate tensile strength increases. This behavior could be accounted for by the increase of the number of entanglements with the increase of molar mass. It should be noted that polylactide polymers created in our work have a much higher creep strength than other polylactide polymers [27,28,29]. The films described in the article were shown to be susceptible to destruction in water solutions even in the absence of biological objects (Figure 5). The less molar mass of polylactide used in polymer film production, the slower the degradation of the polymer. Polymer degradation occurs in two phases. The first, rapid, phase is mostly related to the swelling and depolymerization of bonds between the initial particles. The second phase is possibly related to the breakdown of the polymer scaffold. Such processes are observed during the thermal degradation of polylactide [30].

Kinetics of prourokinase release from polylactide-based membranes were shown to depend on the immobilized substance concentration in the membrane and polylactide molar mass (Figure 7). The more the initial amount of substance in the membrane, the more its release rate per time unit on the initial step. Notably, by the 30th day of experiment, around 50% of initial enzyme molecules remained encapsulated in the membrane. The other 50% were released from the membrane to the environment.

Biodegradable polymer membranes studied in the current work are suitable for producing stent coatings and implants with a prolonged and controlled release of medications into surrounding tissues. Many attempts have been made in the recent two decades to immobilize various substances in membranes [31,32,33], but there are still no examples of the controlled release of substances heavier than 10 kDa from the membrane [34]. In the present study, a controlled release of 46 kDa substance was achieved.

The biocompatibility of polymer films produced of polylactide of different molar mass was studied in the work on in vitro models (Figure 8, Figure 9 and Figure 10). Polylactide films were shown to be a preferable substrate for the cells than cultural glassware. A high biocompatibility of polylactide does not cause any doubts [35]. It was interesting to find that the addition of 2% of prourokinase increased the biocompatibility of the polymer. It should be noted that unusual concentration dependencies in the case of use of prourokinase were also found earlier [36,37,38]. The results of cell model experiments were validated on animal models (Figure 11). The data obtained on implantation models showed that all the tested materials are non-toxic for the recipient’s organism and demonstrate high biocompatibility. Analogous results were obtained in the study of polymer samples deposited on stent blanks. Thus, various biodegradable polylactide-based polymer membranes were created and developed in this work, they met the requirements for mechanical properties, and were capable of prolonged targeted release of biomacromolecules of high molecular weight.
